# Supplementary material for: Assessing in vivo mutation frequencies and creating a high-resolution genome-wide map of fitness costs of Hepatitis C virus
Source: PLoS Genet. 2022 May 2;18(5):e1010179. doi: 10.1371/journal.pgen.1010179 (PMC9113599; doi:10.1371/journal.pgen.1010179)
Supplement: S1 Fig — (PDF) [file pgen.1010179.s001.pdf]

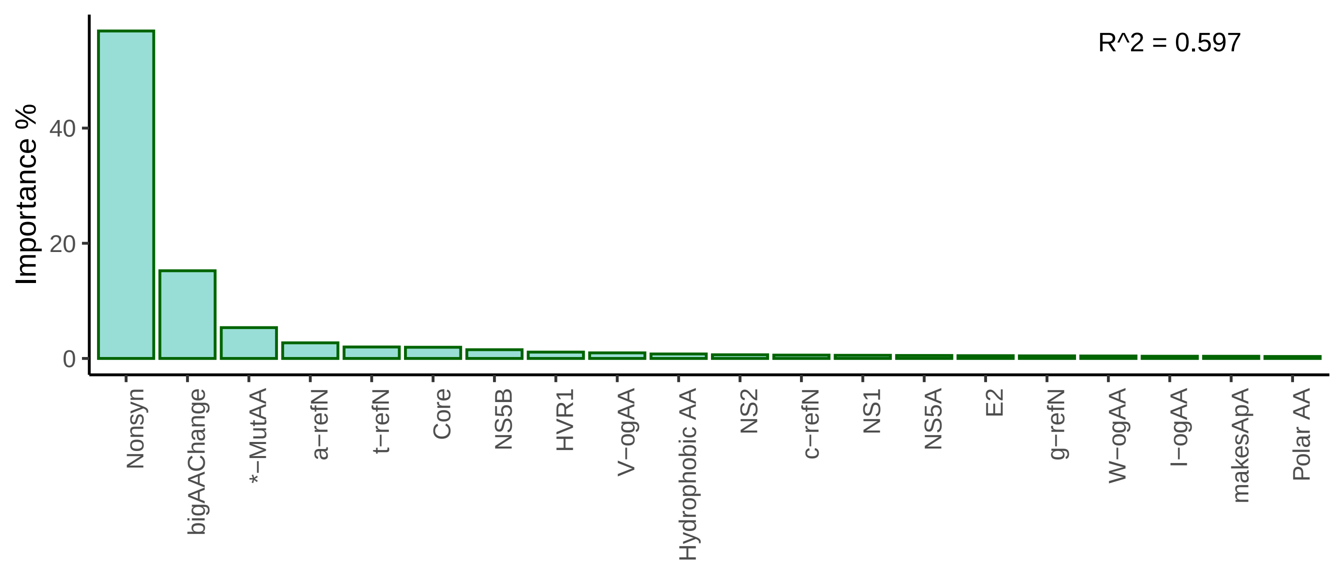
R^2 = 0.597
Importance %
0
20
40
Nonsyn
bigAAChange
*−MutAA
a−refN
t−refN
Core
NS5B
HVR1
V−ogAA
Hydrophobic AA
NS2
c−refN
NS1
NS5A
E2
g−refN
W−ogAA
I−ogAA
makesApA
Polar AA
