# Supplementary figures and images for: Assessing in vivo mutation frequencies and creating a high-resolution genome-wide map of fitness costs of Hepatitis C virus
Source: PLoS Genet. 2022 May 2;18(5):e1010179. doi: 10.1371/journal.pgen.1010179 (PMC9113599; doi:10.1371/journal.pgen.1010179)

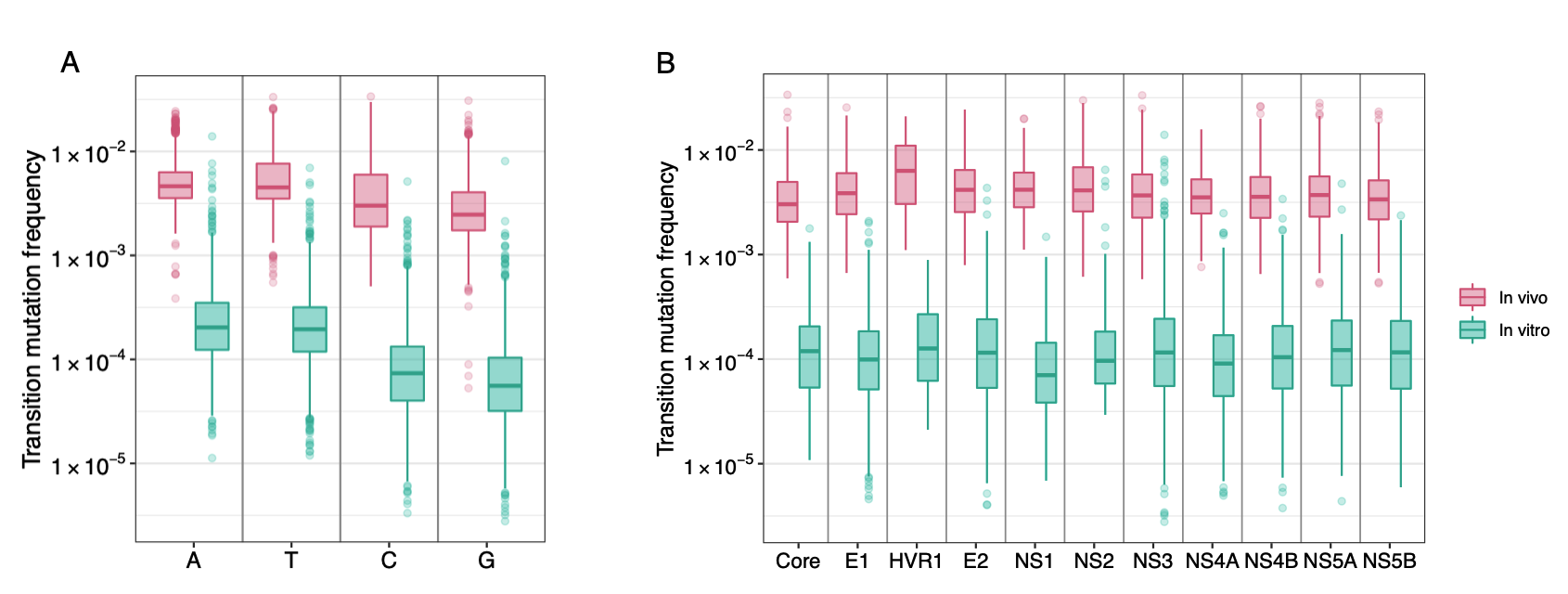

Supplement: S2 Fig — (PNG) [file pgen.1010179.s002.png]

Transition mutation frequency

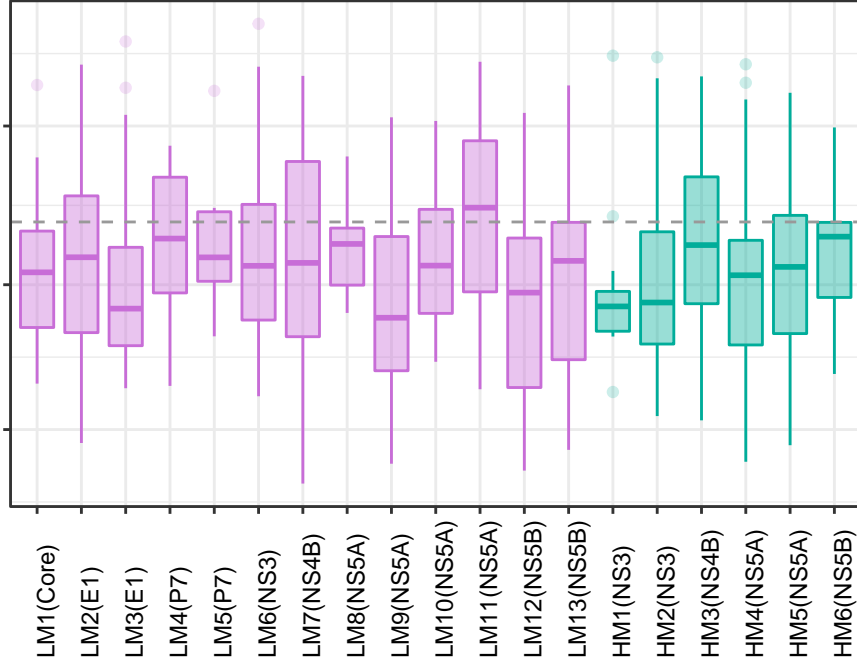

Type

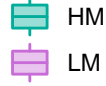

Supplement: S3 Fig — (PDF) [file pgen.1010179.s003.pdf]

Estimated mutation rate  $\pm$  95% CI

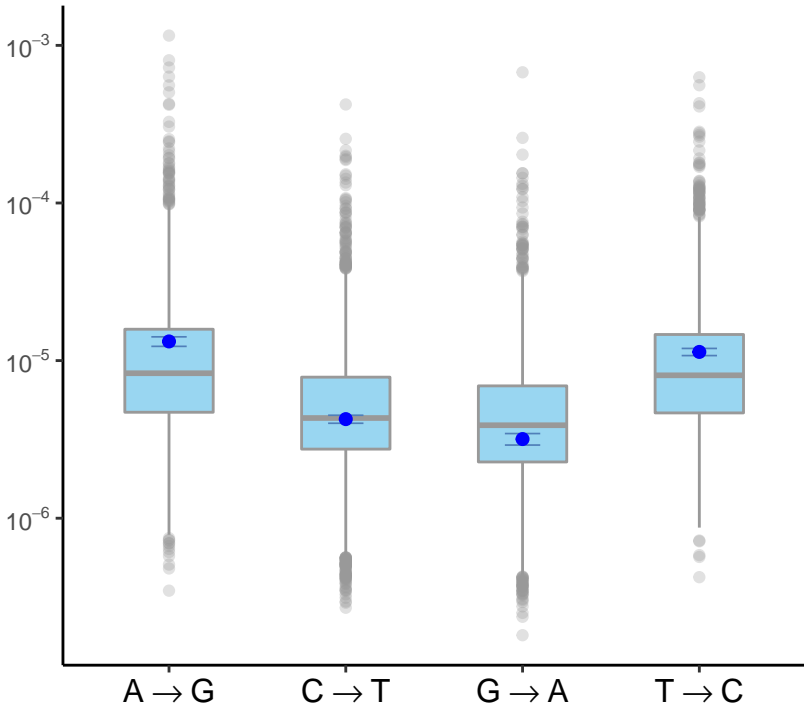

Supplement: S4 Fig — (PDF) [file pgen.1010179.s004.pdf]

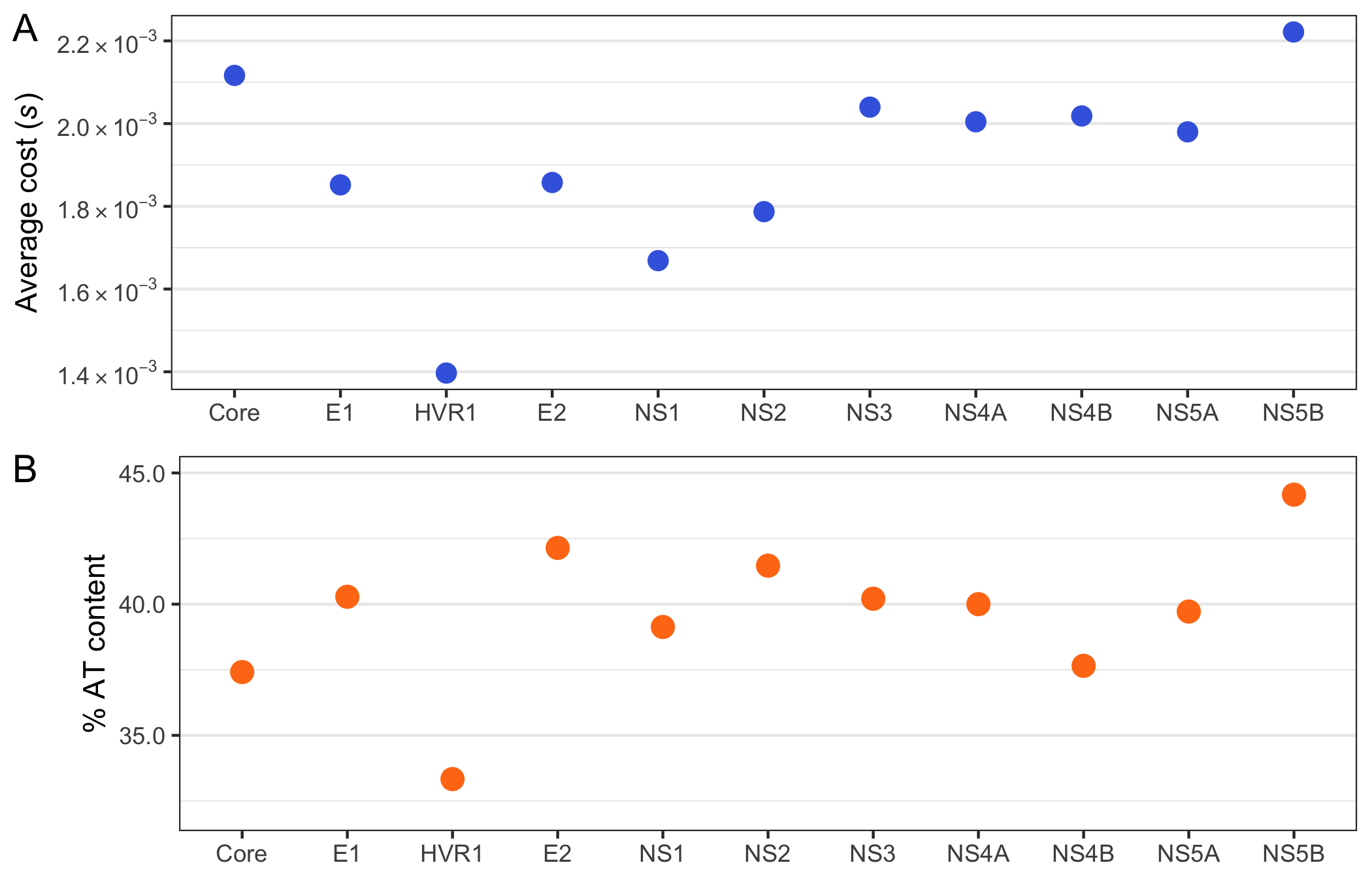

Supplement: S5 Fig — (PNG) [file pgen.1010179.s005.png]

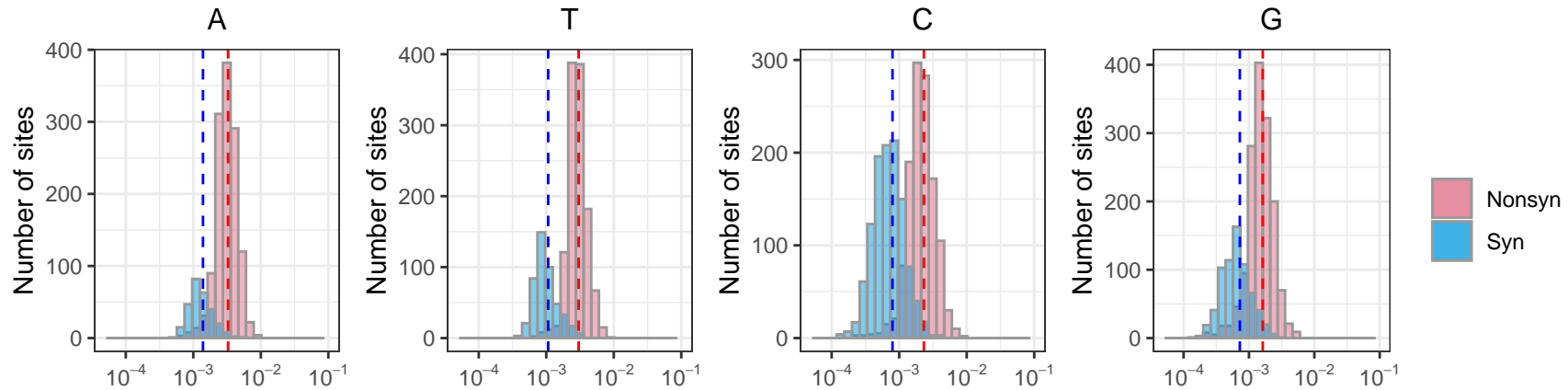

Supplement: S6 Fig — (PDF) [file pgen.1010179.s006.pdf]

Percent

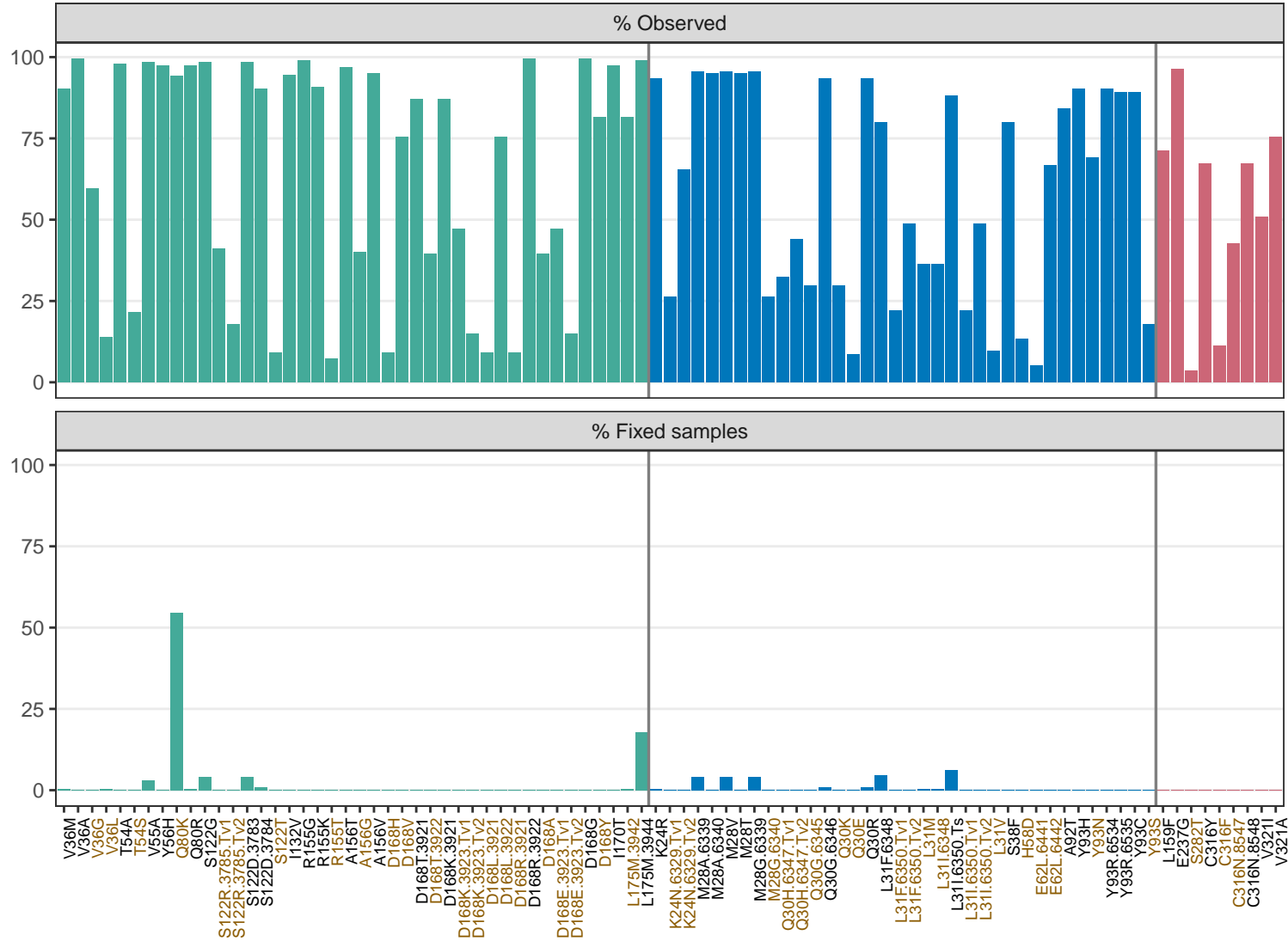

Supplement: S7 Fig — (PDF) [file pgen.1010179.s007.pdf]

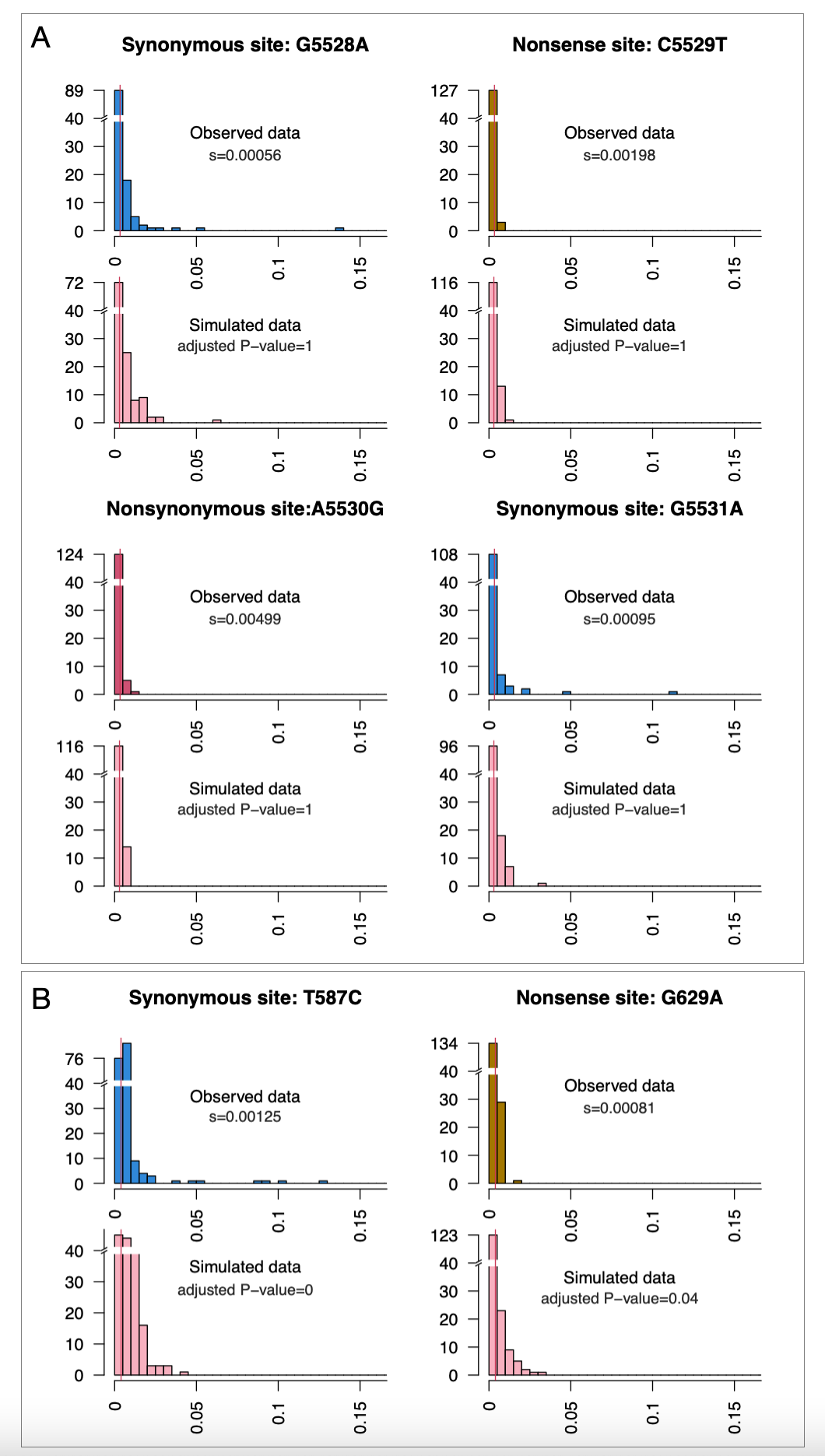

Supplement: S8 Fig — (PNG) [file pgen.1010179.s008.png]

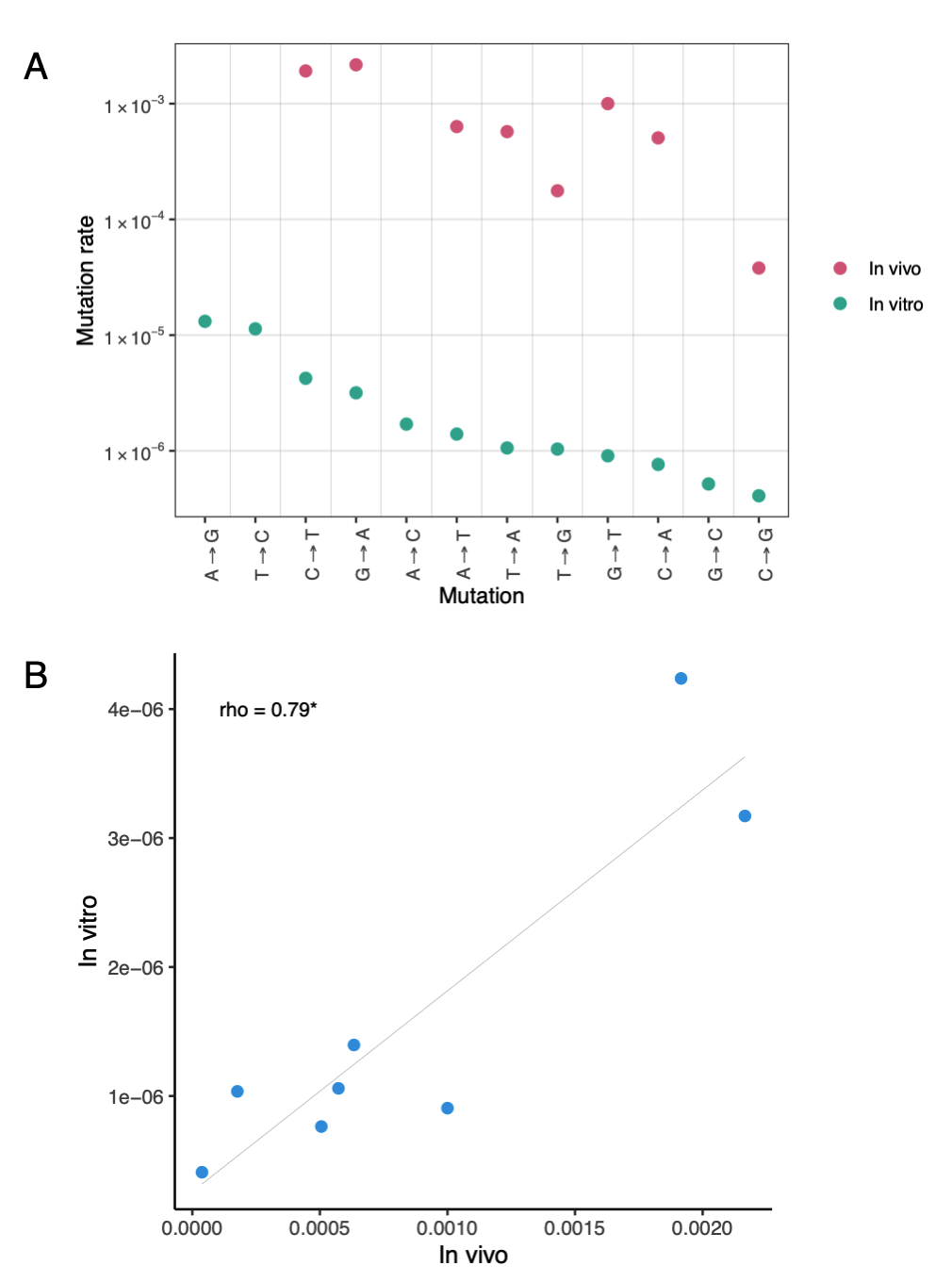

Supplement: S9 Fig — Correlation between estimated in vivo and in vitro mutation rates are shown in B. (PNG) [file pgen.1010179.s009.png]

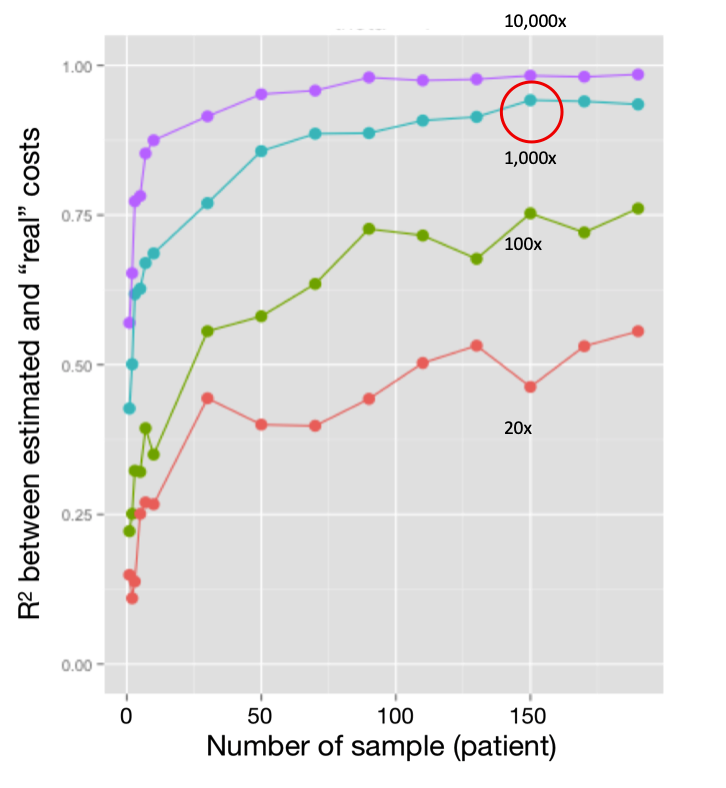

Supplement: S10 Fig — (PNG) [file pgen.1010179.s010.png]

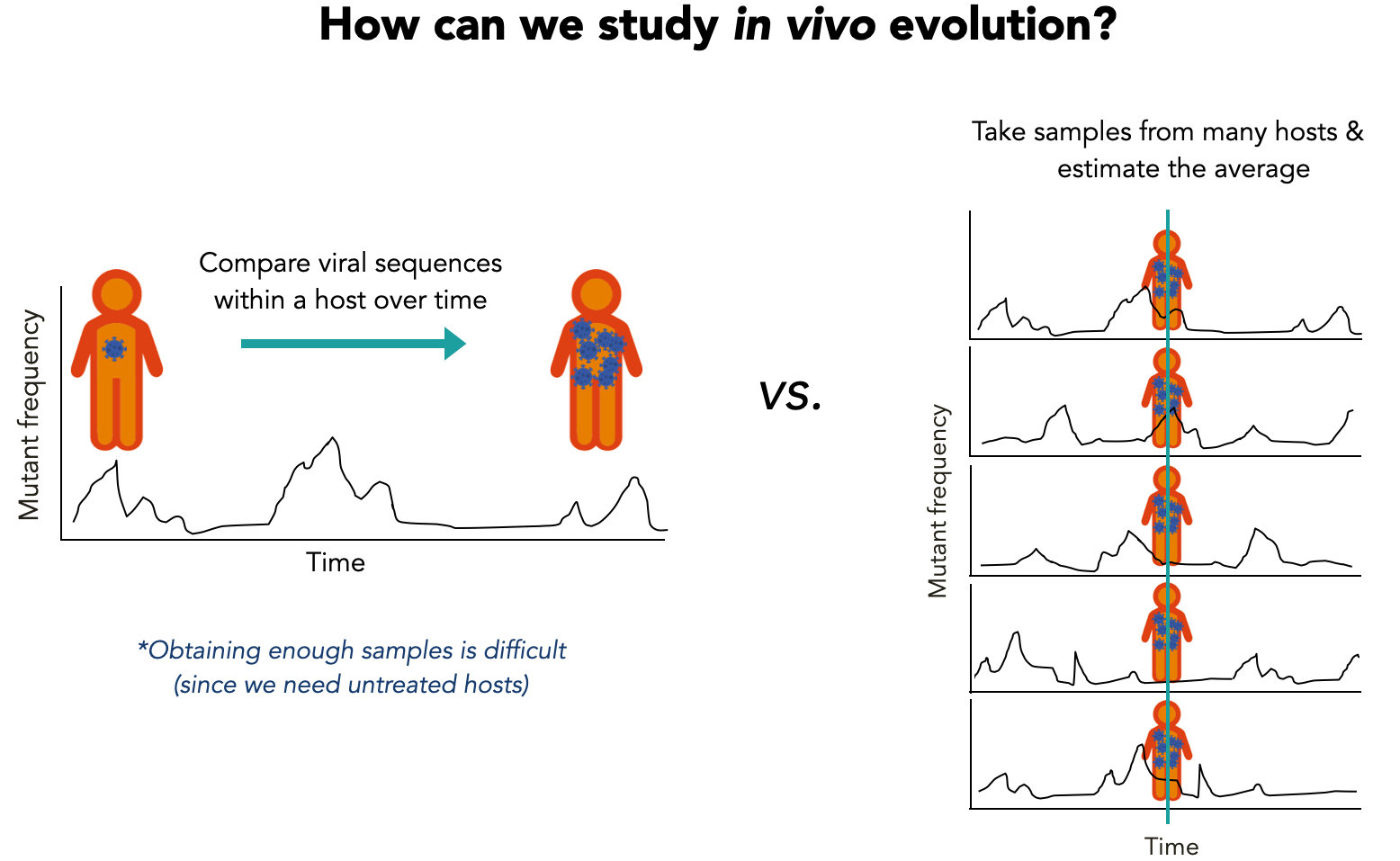

Supplement: S11 Fig — (PNG) [file pgen.1010179.s011.png]
