# Supplementary material for: Assessing in vivo mutation frequencies and creating a high-resolution genome-wide map of fitness costs of Hepatitis C virus
Source: PLoS Genet. 2022 May 2;18(5):e1010179. doi: 10.1371/journal.pgen.1010179 (PMC9113599; doi:10.1371/journal.pgen.1010179)
Supplement: S1 Text — (PDF) [file pgen.1010179.s019.pdf]

Supporting Information (S1 Text) File:

S1 Text: Validation of our frequency based methods: Between-host variability is consistent with within-host variability in HCV.

We assessed the relationship between genetic variability within hosts (*in vivo*) and between hosts by 1) testing the existence of correlation between the within-host and between-host nucleotide diversity at each nucleotide site (assuming that positive correlation exists), 2) checking the proportion of excluded sites through our filtering process to make sure that we did not eliminate too many sites/samples from our analysis to bias our results, and 3) assessing that the highly conserved sites between-host were also conserved within-host to further confirm that patterns of variability are consistent between-host and within-host.

First, we assessed nucleotide diversity levels between- and within-host using (1) average minor variant frequencies to represent within-host (*in vivo*) diversity and (2) Shannon's entropy indices of the HCV genome sequences downloaded from NCBI GenBank (n=423) along with consensus sequences of each viral population (*i.e.* each patient) from our dataset to represent between-host diversity. The two measures of diversity showed a high correlation with Spearman's $\rho = 0.691$ (95% confidence interval =0.6776 - 0.7040, $P < 2.2\text{x } 10^{-16}$) (Fig A), indicating that genome-wide evolutionary patterns were consistent between within-host and between-host. To put this correlation value ($\rho = 0.69$) into a context, we also calculated ρ values for comparisons between different subtypes. When within-host diversity of this study (HCV1a) was compared to between-host diversity of HCV subtype 1b (n=268) and 3a (n=540), ρ values decreased to 0.582 for subtype 1b and 0.527 subtype 3a, as expected (*i.e.* it was not a random association).

Next, we investigated potential effects of our filtering process, where we excluded the sites that had a majority nucleotide different from the reference nucleotide (H77) from each viral population (see Methods). If too many sites were excluded, and if these sites had high variability, this could mask within-host variability. We first calculated the proportion of excluded sites due to the nucleotide difference for each viral population, which was on average, only 6.6 (± 0.1) %. We also compared the consensus (majority nucleotide) sequences of all viral populations to gain further insights into the genetic variability between hosts. The overall sequence similarity (the average pairwise identity of consensus sequences) was relatively high (91.8%), and was within the range of the expected values (> 90% within a subtype) [1–3]. These results indicate that the number of sites excluded from the analysis was quite small, and the filtering step probably did not bias our results.

Lastly, we investigated if a group of highly conserved sites between-host also corresponded to highly conserved sites within-host. Alignments of all consensus sequences across the 195 genomes showed 49% of sites to be identical within the HCV coding regions. These identical sites, or highly conserved sites, occurred relatively evenly throughout the genome, except for in the HVR1 region, which had zero identical sites across the populations. Relative to non-identical sites, identical sites had a significantly lower (-5%) average mutation frequency ($4.69 \text{ x } 10^{-3}$ *vs*. $4.93 \text{ x } 10^{-3}$, Mann-Whitney test, $P = 0.0003$) and a significantly higher (+4%) average selection coefficient ($2.68 \text{ x } 10^{-3}$ *vs*. $2.58 \text{ x } 10^{-3}$, Mann-Whitney test, $P = 0.032$), confirming the correspondence between conserved sites between-host and within-host. Additionally, our recent study showed that close to 60% of sites in the HCV genome were

conserved not only within subtypes but also between different genotypes/subtypes [4], indicating that the majority of sites are evolutionarily conserved (*i.e.* in mutation-selection balance).

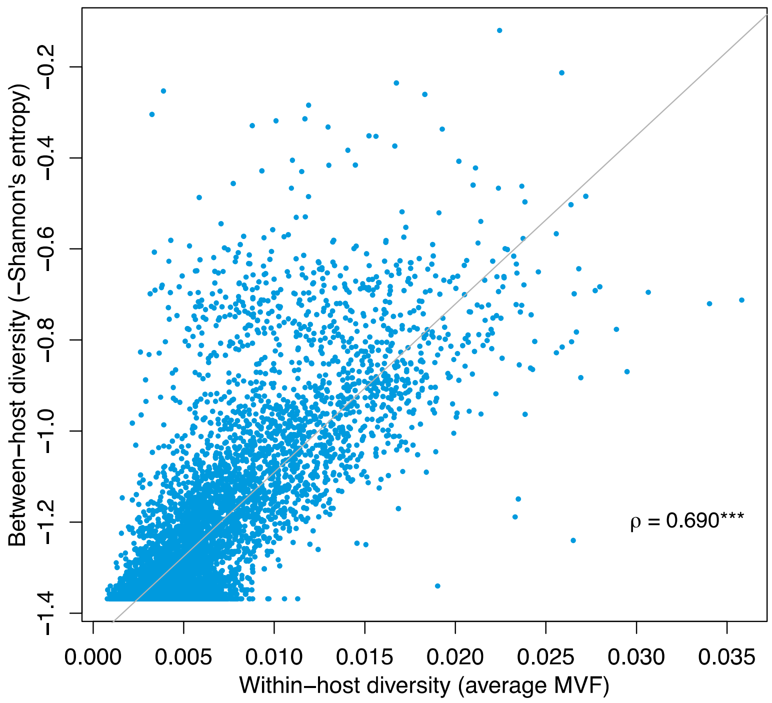

Fig A. Within-host (*in vivo*) *vs*. between-host nucleotide diversity comparison. Between host diversity (Shannon's entropy) at each site was calculated from 618 HCV1a genome sequences (obtained from NCBI GenBank and the consensus sequences generated from each patient sample from this study). For within-host diversity, average minor variant frequencies (MVFs) of all samples at each site were used.

References:

1. Caudill VR, Qin S, Winstead R, Kaur J, Tisthammer K, Pineda EG, et al. CpG-creating mutations are costly in many human viruses. Evol Ecol. 2020;34: 339–359. doi:10.1007/s10682-020-10039-z

2. Echeverría N, Betancour G, Gámbaro F, Hernández N, López P, Chiodi D, et al. Naturally occurring NS3 resistance-associated variants in hepatitis C virus genotype 1: Their relevance for developing countries. Virus Research. 2016;223: 140–146. doi:10.1016/j.virusres.2016.07.008

3. Keck Z, Girard-Blanc C, Wang W, Lau P, Zuiani A, Rey FA, et al. Antibody Response to Hypervariable Region 1 Interferes with Broadly Neutralizing Antibodies to Hepatitis C Virus. Ou JHJ, editor. Journal of Virology. 2016;90: 3112–3122. doi:10.1128/JVI.02458-15

4. Tisthammer KH, Dong W, Joy JB, Pennings PS. Comparative Analysis of Within-Host Mutation Patterns and Diversity of Hepatitis C Virus Subtypes 1a, 1b, and 3a. Viruses. 2021;13: 511. doi:10.3390/v13030511
