# Supplementary material for: Assessing in vivo mutation frequencies and creating a high-resolution genome-wide map of fitness costs of Hepatitis C virus
Source: PLoS Genet. 2022 May 2;18(5):e1010179. doi: 10.1371/journal.pgen.1010179 (PMC9113599; doi:10.1371/journal.pgen.1010179)
Supplement: S2 Text — (PDF) [file pgen.1010179.s020.pdf]

S2 Text: Supplementary Methods: Validating the estimated mutation rates.

The mutation rates estimated from Geller et al. [1] from the *in vitro* condition should not show the effects of different types of mutations, such as synonymous and nonsynonymous mutations. To validate this, we investigated the nucleotide-level transition mutation rates estimated from Geller et al. [1] using a beta-regression model, where the effects of different factors (types of mutations, location, RNA structure) on mutation rates were assessed. Since the dataset contained many zeros, a transformation [y = x (n − 1) + 0.5)/n] (n = the sample size) were applied as recommended by [2]. The best fit model is shown below, which revealed no significant effects of nonsynonymous mutations, CpG creating mutations, and HVR1, as expected from the *in vitro* condition without selective pressures from the host's immune systems. The largest effects were due to the type of ancestral nucleotide, with the mutations rates of C→T and G→A being about a half of that of T→C and A→G. However, the model results show small effects of mutations that cause drastic amino acid change (bigAAChange) and locations (E1, NS1, NS2, NS5B), which we have no explanation for.

	Estimate	Z-value	*P*-value	Effects
(Intercept)	-8.0072	-512.50	<0.0001	0%
t	-0.0438	-2.471	0.0135	-4.3%
c	-0.5938	-32.163	<0.0001	-44.8%
g	-0.7159	-37.205	<0.0001	-51.1%
bigAAChange	0.0372	2.815	0.0049	3.8%
Core	-0.0604	-1.991	0.0464	-5.9%
E1	-0.0737	-2.6837	0.0073	-7.1%
NS1	-0.1961	-3.9992	0.0001	-17.8%
NS2	-0.1938	-7.1382	<0.0001	-17.6%
NS5B	-0.0596	-3.5023	0.0005	-5.8%

To further assess the validity of the estimated mutation rates from Geller et al. [1], we estimated selection coefficients using mutation rates calculated at different levels; 1) an individual site-based level and 2) a quartile-based level, and compared to those estimated using a single, uniform mutation rate for each nucleotide used in the main analysis. The site-based mutation rates were calculated separately for every site along the genome. The quartile-based mutation rates were calculated by grouping the mutation rates into quartiles and averaging them. The results showed that the original method using a uniform mutation rate for each ancestral nucleotide produced the narrowest distributions of selection coefficients, compared to the other two methods for all four nucleotides, indicating the best approach. Examples from C→T mutations are shown in the figure below (Fig A)

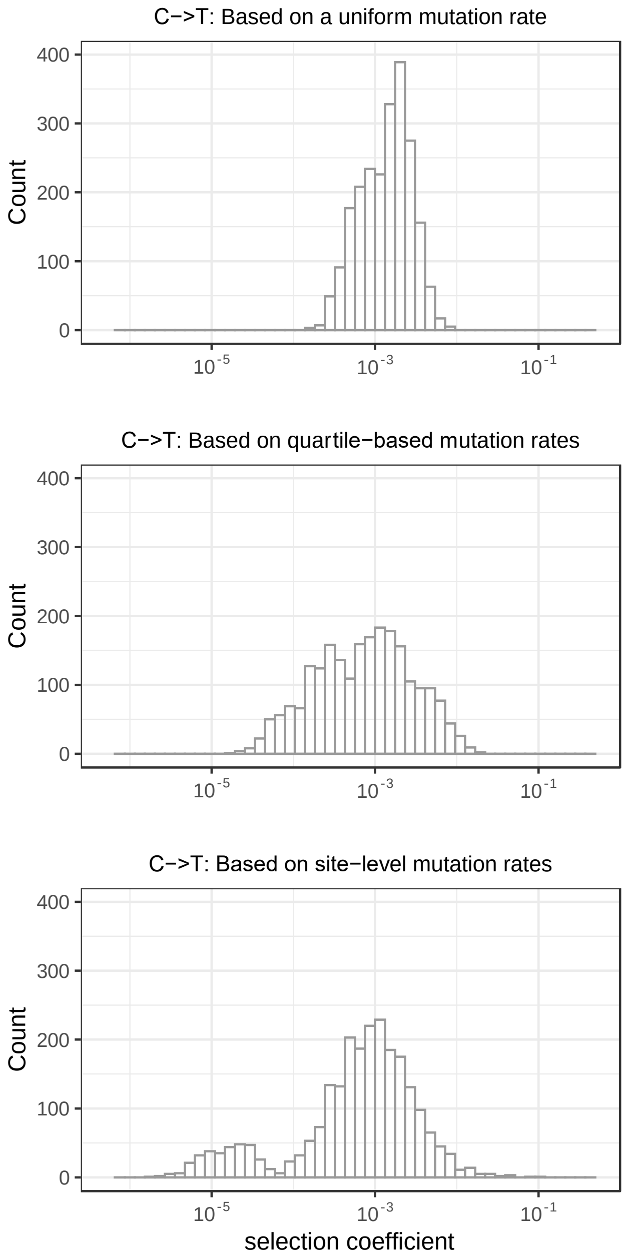

Fig A. Estimated selection coefficients using different levels of mutation rates.

References:

1. Geller R, Estada Ú, Peris JB, Andreu I, Bou J-V, Garijo R, et al. Highly heterogeneous mutation rates in the hepatitis C virus genome. 2016;1: 16045. doi:10.1038/nmicrobiol.2016.45

2. Cribari-Neto F, Zeileis A. Beta Regression in R. Journal of Statistical Software. 2010;34: 1–24. doi:10.18637/jss.v034.i02
