# Supplementary material for: Assessing in vivo mutation frequencies and creating a high-resolution genome-wide map of fitness costs of Hepatitis C virus
Source: PLoS Genet. 2022 May 2;18(5):e1010179. doi: 10.1371/journal.pgen.1010179 (PMC9113599; doi:10.1371/journal.pgen.1010179)
Supplement: S12 Fig — (PDF) [file pgen.1010179.s012.pdf]

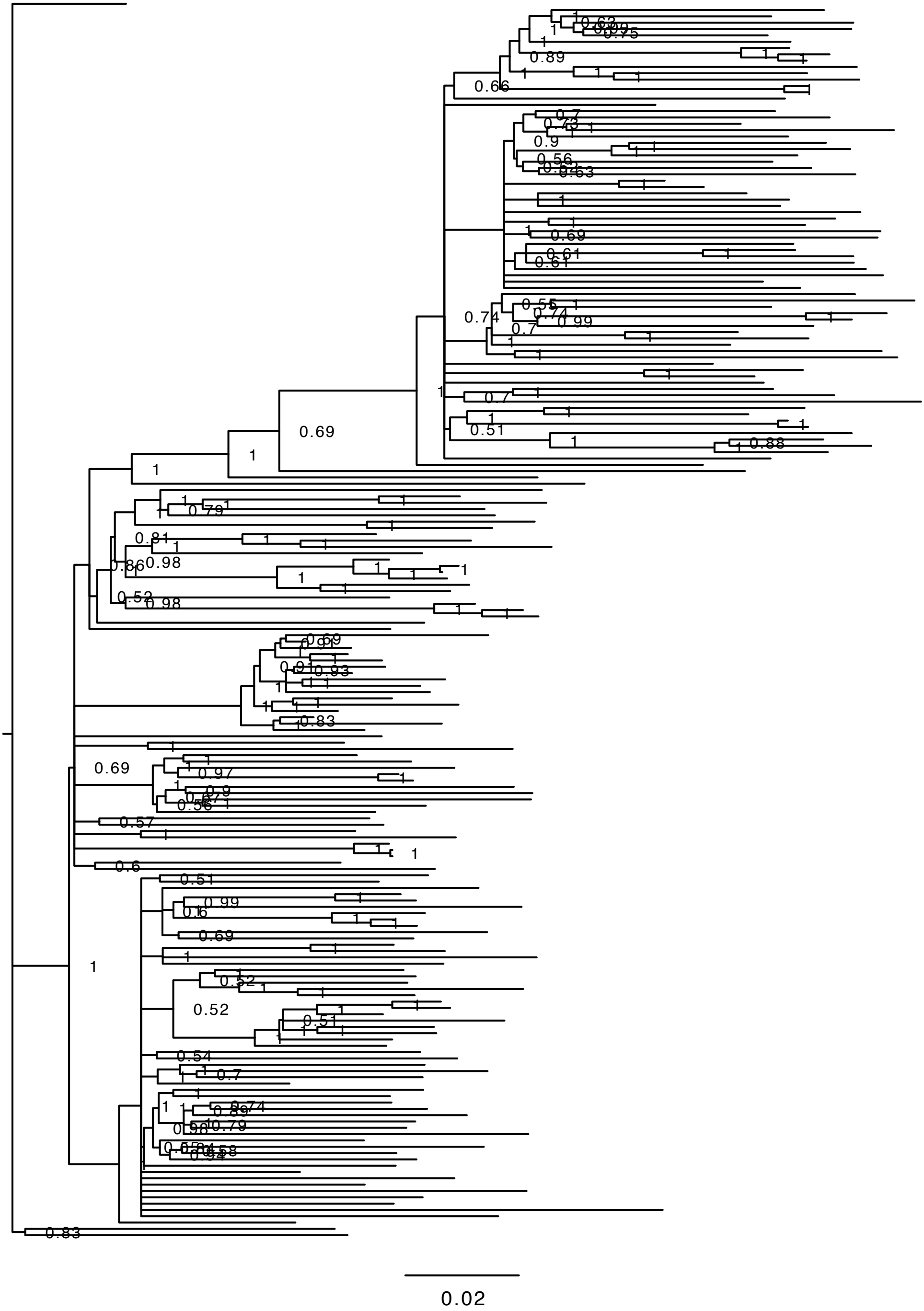
0.02
